# *Rickettsia felis* and Changing Paradigms about Pathogenic Rickettsiae

**DOI:** 10.3201/eid2010.131797

**Published:** 2014-10

**Authors:** Marcelo B. Labruna, David H. Walker

**Affiliations:** University of São Paulo, São Paulo, Brazil (M.B. Labruna);; University of Texas Medical Branch, Galveston, Texas, USA (D.H. Walker)

**Keywords:** Rickettsia felis, asymptomatic infection, chronic infection, seronegative infection, vector-borne infections, malaria

**To the Editor:** Mediannikov et al. recently reported several features common to the epidemiology of *Rickettsia felis* infection and malaria in Africa (*1*). Similar to the findings of several other recent studies in Africa (*2*,*3*), the authors diagnosed *R. felis* infection in febrile—and to a lesser extent in afebrile—persons by detecting *R. felis* DNA in human blood samples processed by highly sensitive real-time PCR. These results challenge some paradigms in rickettsiology that need to be more critically evaluated.

Because *R. felis* DNA was detected in circulating blood of asymptomatic persons (albeit more frequently in patients with mild febrile illness), Mediannikov et al. proposed that humans could be a natural reservoir of *R. felis*, as they are for malaria parasites. *R. felis* antibodies failed to develop in nearly all patients in whom *R. felis* DNA was detected, even after repeated detection of *R. felis* DNA. In 2 other studies, the same researchers proposed that patients might have several episodes of *R. felis* infection (relapse or reinfection) to explain why DNA of the agent was detected in the blood at multiple times (*2*,*3*). They also proposed that the absence of an antibody response would explain why the disease relapses in some persons (*3*).

These changing paradigms in rickettsiology require thorough evaluation. Once inside a vertebrate host, pathogenic rickettsiae have been believed to multiply primarily within endothelial cells in the patient’s organs. As far as we know, rickettsiae do not multiply within circulating blood cells (*4*). In contrast, the agents of malaria (*Plasmodium* spp.) are typically parasites of erythrocytes. Therefore, a blood sample from a person with malaria is an excellent source for PCR diagnostic testing. The sensitivity of PCR for rickettsiae in human blood samples is very low because the sensitivity depends on the magnitude of the vasculitic lesions, i.e., the number of endothelial cells destroyed or detached by rickettsial growth, resulting in circulating rickettsiae. *R. conorii* (*5*) and *R. rickettsii* (*6*) were detected by highly sensitive PCR in 100% of fatal cases and in only very few nonfatal cases.

In addition to never having been isolated from humans, *R. felis* has many characteristics of a symbiotic organism. It possesses a mosaic structure genome (size 1.48 Mb) with a high coding capacity (83%) that is typical of symbiotic bacteria (*7*). Merhej et al. have proposed that within a given bacterial genus (including *Rickettsia*), pathogenic species have smaller genomes than nonpathogenic species (*8*). In the genus *Rickettsia,* the pathogens *R. rickettsii*, *R. prowazekii*, *R. sibirica*, *R. typhi*, *R. parkeri*, and *R. conorii* have genomes of ≈1.2–1.3 Mb, whereas the apparently nonpathogenic *R. bellii* has a 1.5-Mb genome, similar to that of *R. felis*. In contrast to the well-known pathogenic *Rickettsia* species, *R. felis* has been reported in a variety of invertebrate hosts, including hematophagous (fleas, ticks, flies, mosquitoes) and non-hematophagous (book lice) arthropods (*9*). Behar et al. have suggested that *R. felis* is responsible for inducing parthenogenesis in book lice, similar to the manner of *Wolbachia* organisms in various invertebrate hosts (*9*). Furthermore, *R. felis* forms mycetomes in book lice, a growth feature typical of bacterial endosymbionts (*10*).

The current view in rickettsiology has a strong anthropocentric bias because the studies have concentrated on parasitic arthropods that feed on humans rather than on free-living arthropods. In fact, the number of *Rickettsia* species associated with non-hematophagous hosts might be much greater than the ones of medical importance (*9*). Thus, considering *R. felis* as an important pathogen in Africa (and in the world) might be premature. Several questions need to be answered before such a conclusion. In asymptomatic persons in whom endothelial cells are likely to be intact, where does *R. felis* grow to be released at detectable levels in the circulating blood? Considering that all classical spotted fever agents induce an antibody response (*4*), why do *R. felis* antibodies fail to develop in humans after a clinical illness attributed to *R. felis*? In addition, repeated reports that the main vector of *R. felis* is the cat flea, *Ctenocephalides felis*, need to be proven by experimental demonstration of its vector capacity.

Given the numerous questions about *R. felis*, we would add another: could *R. felis* be a symbiont of a human parasite, such as a protozoon or a helminth? Obviously, the answer is unknown. However, had we not known that *Wolbachia* organisms are typically endosymbiotic bacteria of both human and animal filarial nematodes, what would we conclude if we detected *Wolbachia* DNA in blood of either asymptomatic or ill patients?
